# A High Prevalence of Anti-EBNA1 Heteroantibodies in Systemic Lupus Erythematosus (SLE) Supports Anti-EBNA1 as an Origin for SLE Autoantibodies

**DOI:** 10.3389/fimmu.2022.830993

**Published:** 2022-02-17

**Authors:** Viktoryia Laurynenka, Lili Ding, Kenneth M. Kaufman, Judith A. James, John B. Harley

**Affiliations:** ^1^Center for Autoimmune Genomics and Etiology, Cincinnati Children’s Hospital Medical Center, Cincinnati, OH, United States; ^2^Division of Biostatistics and Epidemiology, Department of Pediatrics, Cincinnati Children’s Hospital Medical Center, Cincinnati, OH, United States; ^3^Department of Pediatrics, University of Cincinnati College of Medicine, Cincinnati, OH, United States; ^4^Research Service, US Department of Veterans Affairs Medical Center, Cincinnati, OH, United States; ^5^Arthritis and Clinical Immunology Program, Oklahoma Medical Research Foundation, Oklahoma City, OK, United States; ^6^Department of Medicine, University of Oklahoma Health Science Center, Oklahoma City, OK, United States; ^7^Department of Pathology, University of Oklahoma Health Science Center, Oklahoma City, OK, United States; ^8^Cincinnati Education and Research for Veterans Foundation, Cincinnati, OH, United States

**Keywords:** systemic lupus erythematosus (SLE), etiology, Epstein–Barr virus (EBV), anti-EBNA1, molecular mimicry, autoantibodies

## Abstract

**Background:**

That Epstein–Barr virus (EBV) infection is associated with systemic lupus erythematosus (SLE) is established. The challenge is to explain mechanistic roles EBV has in SLE pathogenesis. Previous studies identify four examples of autoantibody cross-reactions between SLE autoantigens and Epstein–Barr nuclear antigen 1 (EBNA1). For two of these examples, the earliest detected autoantibody specifically cross-reacts with EBNA1; thereby, defined EBNA1 epitopes induce a robust autoantibody response in animals. These results suggest that the autoantibodies initiating the process leading to SLE may emerge from the anti-EBNA1 heteroimmune response. If this hypothesis is true, then anti-EBNA1 responses would be more frequent in EBV-infected SLE patients than in EBV-infected controls. We tested this prediction.

**Methods:**

We evaluated published East Asian data by selecting those with a positive anti-viral capsid antigen (VCA) antibody immunoglobulin G (IgG) test and determining whether anti-EBNA1 was more common among the EBV-infected SLE cases than among matched EBV-infected controls with conditional logistic regression analysis.

**Results:**

All the qualifying SLE patients (100%) in this dataset were EBV-infected compared to age- and sex-matched controls (92.2%) [odds ratio (OR) = 28.6, 95% CI 6.4–∞, p = 8.83 × 10^-8^], confirming the known close association of EBV infection with SLE. Furthermore, virtually all the SLE cases have both anti-VCA IgG and anti-EBNA1 IgG antibodies [124 of 125 (99.2%)], which are more frequently present than in age- and sex-matched EBV-infected controls [232 of 250 (93.2%)] (OR = 9.7, 95% CI 1.5–414, p = 0.0078) for an 89.7% SLE attributable risk from anti-EBNA1, which is in addition to the 100% SLE risk attributable to EBV infection in these data.

**Conclusions:**

The association of EBV infection with SLE is reconfirmed. The prediction that anti-EBNA1 is more frequent in these SLE cases than in EBV-infected controls is true, consistent with the hypothesis that anti-EBNA1 contributes to SLE. This second EBV-dependent risk factor is consistent with a molecular mimicry model for the generation of SLE, starting with EBV infection, progressing to anti-EBNA1 response; then molecular mimicry leads to anti-EBNA1 antibodies cross-reacting with an SLE autoantigen, causing autoantibody epitope spreading, and culminating in clinical SLE. These results support the anti-EBNA1 heteroimmune response being a foundation from which pathogenic SLE autoimmunity emerges.

## Introduction

Evidence implicating Epstein–Barr virus (EBV) in the pathogenesis of systemic lupus erythematosus (SLE) is compelling (1–7). As a mechanistic component, the immune response against EBV Epstein–Barr nuclear antigen 1 (EBNA1) has been identified as a candidate for the heteroimmune response from which pathogenic lupus autoimmunity arises *via* cross-reactivity with anti-Sm B/B’, anti-Sm D, anti-Ro, and, recently, anti-C1q (8–13). If this phenomenon is general and the anti-EBNA1 heteroimmune antibody response, in all of its complexity, is the substrate for the generation of SLE autoantibodies, then anti-EBNA1 antibody responses, which are found in 70% to 90% of EBV-infected persons, would be present at a higher rate in SLE patients. A hint that this may be true was previously found in a small pediatric cohort, where this antibody was present in 69% of the matched normal EBV-infected controls and in 100% of the SLE cases, all of whom were also EBV-infected [odds ratio (OR) >30, p < 0.001] (14). We sought to test the generality of this finding using independent data, given its potentially important implications for identifying the origins of SLE autoimmunity.

EBNA1 is an unusual immunogen and antigen (15). Perhaps, these properties are potential contributing factors to the possible anti-EBNA1 origin of SLE autoimmunity. There are many fewer anti-EBNA1-specific CD8 T cells than expected after EBV infection, despite EBNA1 being expressed in virtually all the canonical latency states of EBV-infected B cells. The lower CD8 response has been attributed to nuclear localization, to proteosome inhibition by the Glycine-Alanine repeat domain of EBNA1, and to the inhibition of EBNA1 mRNA translation by Guanine-quadraplexes (16–22). The humoral consequences of the unusual features EBNA1 immunogenicity have not been evaluated. A small study (14) also identified anti-EBNA1 fine specificity differences between pediatric SLE patients and controls.

Cui et al. (1) provide an independent dataset appropriate to test the prediction that anti-EBNA1 is increased in SLE. They reported anti-EBV viral capsid antigen (VCA) IgG serological studies ordered by practicing physicians for 6,289 patients from their clinical laboratory in Beijing. These real-world data use as entry criteria the clinical decision to evaluate anti-VCA serology and rely upon the choices and diagnoses of practicing physicians. The data show that EBV infection is more frequent in SLE than in the other EBV-tested patients who are not diagnosed with SLE (1), consistent with previous studies (2–7). Anti-EBNA1 IgG is virtually always also present in these SLE patients in contrast to the lower rate in controls with other diagnoses, supporting not only EBV as an important contributor to the etiology of SLE but also potentially operating through the hypothetical mechanism that SLE humoral autoimmunity arises from the anti-EBNA1 heteroimmune response.

## Methods

### Patients

Cui et al. (1) ascertained subjects upon the clinical decision to evaluate EBV serology. For this analysis, we concentrate on 6,289 subjects tested for anti-EBV VCA IgG in blood [see **Supplementary Table** in “**S1 File**” in (1)] as the most reliable indicator of prior EBV infection. Except for one newborn, the youngest SLE patient was 6 years old; therefore, this case of possible neonatal lupus, along with controls less than 6 years old, was removed from the analysis. In addition, following the decisions of Cui et al. (1), we removed those with equivocal anti-VCA IgG test results; all 50 of these were controls, leaving 5,803 patients of all diagnoses and situations with results to address the question posed. Of the 424 SLE patients in the database, these steps resulted in 232 SLE patients qualifying for analyses. No private identifying information was available from any subject to the authors of this study, and no interaction occurred between the subjects and the authors.

### Serological Testing

EBV serological tests manufactured by Euroimmun (Lübeck, Germany) were used in Cui et al. (1), who followed the manufacturer’s instructions for anti-VCA for IgM, IgG, and IgA, anti-EBNA1 IgG, and anti-Early Antigen Diffuse (EA/D) IgG.

### Data Analysis

Logistic regression models were used to examine associations between anti-EBV antibodies (anti-VCA IgG and anti-EBNA1 IgG) and SLE status among anti-VCA IgG-tested and anti-VCA IgG-positive subjects, with adjustment for age and sex. The non-linear age effect was investigated by polynomial terms of age. Firth logistic regression was used for situations where an antibody was positive in all or virtually all SLE cases (quasi-complete separation).

The potentially confounding influence of sex and age was addressed by randomly matching 2 or 3 controls by sex and age to each case, without replacement. For age, the great majority of controls (≥98%) were matched to cases within the same year of age. For three exceptions, older and younger controls of 1–3-year age differences were randomly selected to complete the case:control matches for matched sets. The case:control matches used in this study are presented in **Table S1**.

Conditional logistic regression was used to examine the association between anti-EBV antibodies and SLE status for age- and sex-matched data. Exact conditional logistic regression was used when any cells formed by the presence of anti-EBV antibodies and SLE status had no observations or were too small. Attributable risk, defined as the proportion of disease attributable to a risk factor in the study sample exposed to that factor within the context of the matched sets, was estimated with 95% confidence limits from ORs as (OR-1)/OR. Sensitivity analysis was conducted where attributable risk was estimated with risk ratio (RR) as (RR-1)/RR when estimated RR was available from the models (**Tables S2**–**S7**).

Since the available controls provided more matches for younger patients and since age had the well-known strong impact upon the probability of EBV infection, the sample was also divided into those <11 years old and those >10 years old and evaluated separately in subsidiary analyses (see **Tables S1**–**S7**). All analyses were conducted in SAS v9.4 (SAS Institute, Cary, NC, USA).

## Results

Our analysis of the Beijing data (1) reconfirms that the prevalence of EBV infection is increased in SLE compared to the controls in this dataset. All 232 of the tested patients diagnosed by their physician with SLE were EBV-infected, as detected by the presence of anti-VCA IgG, while 175 of 5,571 of the controls were not EBV-infected by this measure. Logistic regression shows that, as expected, both sex and age are important variables contributing to the differences between SLE cases and controls, respectively, OR = 6.02 (95% CI 4.13–8.75), p = 6.5 × 10^-21^ and OR = 0.93 (95% CI 0.92–0.94), p < 1 × 10^-24^ (**Table S5**), which means that the OR difference between cases and controls decreases by an average of 0.93 for every 1-year increase in age. Sex and age, thereby, become potential confounders in assessing the serologic importance of anti-EBNA1 IgG. Meanwhile, the association of SLE with the presence of anti-EBNA1 antibodies has a large effect without considering the influence of sex, age, or EBV anti-VCA IgG serologic status, OR = 17.6 (95% CI, 3.42–90.4), p = 0.0006 (**Table S5**).

### Anti-VCA IgG Case: Control Matching

To remove the influence of age and sex and isolate the impact of EBV serology, we, therefore, reorganized and reduced the data records considered. We matched controls on age and sex to cases in a 3:1 ratio, resulting in a dataset composed of 232 cases and 696 controls. Overall, a strong association of EBV infection with SLE is present, as measured by anti-VCA IgG (OR = 28.59 (95% CI 6.42–∞), p = 8.83 × 10^-8^) (**Table 1**). The fraction of SLE attributable to EBV infection in these data is complete at 100%, suggesting that all these SLE cases are potentially related to EBV infection. Anti-EBNA1 antibodies are also more prevalent in the SLE cases than in matched controls [OR = 19.07 (95% CI 3.09–789.48), p = 1.82 × 10^-5^] (**Table S2**). Similar results have been obtained in previously evaluated datasets (2, 6, 7, 14). However, virtually all of these studies save one, our previous evaluation of pediatric SLE (14), did not separate the evidence that EBV infection is a risk factor for SLE from any other statistically independent contribution.

**Table 1:** Association between SLE status and anti-EBV antibodies in age- and sex-matched cases and controls.

Anti-EBV antibodies^1^		SLE^5^	Controls	Conditional logistic regression^2^		Attributable fraction^3^
				OR (95% CI)	p^4^	
Anti-VCA IgG	POS	232	642	28.59 (6.42–∞)	8.83 × 10^-8^	100%
	NEG	0	54		
Anti-EBNA1 IgG in Anti-VCA IgG POS	POS	124	232	9.74 (1.49–414.34)	0.0078	89.7%
	NEG	1	18		
Anti-VCA IgA in Anti-VCA IgG POS	POS	72	56	3.47 (2.27–5.29)	5.7 × 10^-8^	71.2%
	NEG	85	258		

^1^Anti-Epstein–Barr virus (EBV) viral capsid antigen (VCA) IgG or IgA and anti-Epstein–Barr nuclear antigen 1 (EBNA1) IgG-positive (POS) or -negative (NEG) test result. Controls are matched to each case by age (6–75 years) and sex.

^2^The exact test is used for anti-VCA IgG and anti-EBNA1 in anti-VCA IgG POS.

^3^The fraction of systemic lupus erythematosus (SLE) cases attributable to the positive antibody result being tested in each instance, estimated from the odds ratio (OR).

^4^One-tailed p values and median unbiased estimates of OR from exact conditional logistic regression are presented for anti-VCA IgG and anti-EBNA1 IgG in anti-VCA IgG POS cases and controls.

^5^The cases and assigned matched controls used in these analyses are presented in **Supplemental Table S1**. Original data are from Cui et al. (1).

In our view, the association of EBV infection with anti-EBNA1 does not address the important question of what are the component risk elements beyond EBV infection that confer risk for SLE. If specific components of EBV are making a contribution to disease risk, then they should be separable from EBV infection by considering their contribution toward SLE risk in isolation, which can be achieved by limiting consideration to only those subjects who are EBV-infected. Since anti-EBNA1 occurs in 70%–90% of EBV-infected controls, this idea can be evaluated by testing for the predicted higher proportion of anti-EBNA1 positivity in the EBV-infected SLE compared to EBV-infected controls. A result showing no difference or a lower frequency of anti-EBNA1 in SLE would contradict the hypothesis, rendering the heuristic proposition false. A result showing that anti-EBNA1 is more frequent in SLE than in controls would fulfill the prediction that follows from the hypothesis, providing additional important circumstantial evidence that the heuristic hypothesis might be true.

For this test, we matched two controls to each SLE case by age and sex, limiting the analysis to 1) the EBV-infected subjects, as determined by an anti-VCA IgG positive test, and 2) those who were tested for anti-EBNA1 and had either a positive or a negative result. We found that positive anti-EBNA1 IgG results were virtually always present in the SLE cases [124 of 125 (99.2%)]; meanwhile, the matched controls did not have anti-EBNA1 as frequently [232 of 250 (92.8%)] [OR = 9.74 (95% CI 1.49–414.34), p = 0.0078] (**Table 1**). The prediction that anti-EBNA1 is more common in EBV-infected SLE than in EBV-infected controls is true in these data with an attributable fraction of 89.7%, consistent with the possibility that the humoral autoimmunity characteristic of SLE generally arises from the anti-EBNA1 heteroimmune response.

Other results in these data provide a comparative perspective for the relationship of EBV infection to SLE. Anti-VCA IgA is reported to be more frequent in SLE than in controls (1, 6). To contrast this association with anti-EBNA1, we selected those subjects who are EBV-infected (anti-VCA IgG positive) and compared the proportion of anti-VCA IgA-positive in the SLE cases [72 of 157 (45.9%)] to matched controls [56 of 314 (17.8%)]. This was also a significant result [OR = 3.47 (95% CI 2.27–5.29), p = 5.7 × 10^-8^] (**Table 1**) but with a much lower estimated attributable fraction at 71.2% than that found with anti-EBNA1 at 89.7%. Matching sex and age for anti-VCA IgM and anti-EA/D IgG (**Table S1**) did not produce significant results. Additional analyses from matched and unmatched data can be found in **Tables S2**–**S7**.

## Discussion

The strong association of EBV infection with SLE in these East-Asian data (1) confirms our previous observations in European and African-American children and adults with SLE (2, 23) and is consistent with previous studies and meta-analyses (3–7). These studies measured EBV infection mostly by serology, though a direct assay of EBV DNA from peripheral blood has also demonstrated an association (2). The conclusion that SLE is associated with EBV infection is convincing. The association of EBV with SLE is intriguing, but by itself does not compel a conclusion of causal etiology, despite the many other abnormalities of the EBV infection found in SLE patients that contribute additional circumstantial evidence consistent with causation [reviewed in (4)]. Additional EBV-dependent features would make the case for causation much stronger.

The anti-EBNA1 antibody response has become a candidate component or SLE risk from EBV infection based on numerous findings, though especially because multiple SLE autoantigens cross-react with EBNA1 heteroimmune antibodies (8–13). For the autoantigens of Sm B/B’ and Ro, the cross-reactive structures occur as the first autoantibodies detected that are formed in the autoimmune response, consistent with being initiating structures of autoimmunity (9, 12). For Sm B/B’, Ro, and C1q, animal models made by immunizing with the cross-reactive structures have manifestations reminiscent of human SLE, demonstrating the potential for systemic autoimmunity from the immune response to the viral-originating EBNA1 cross-reactive structures (12, 24–28).

**Figure 1:**
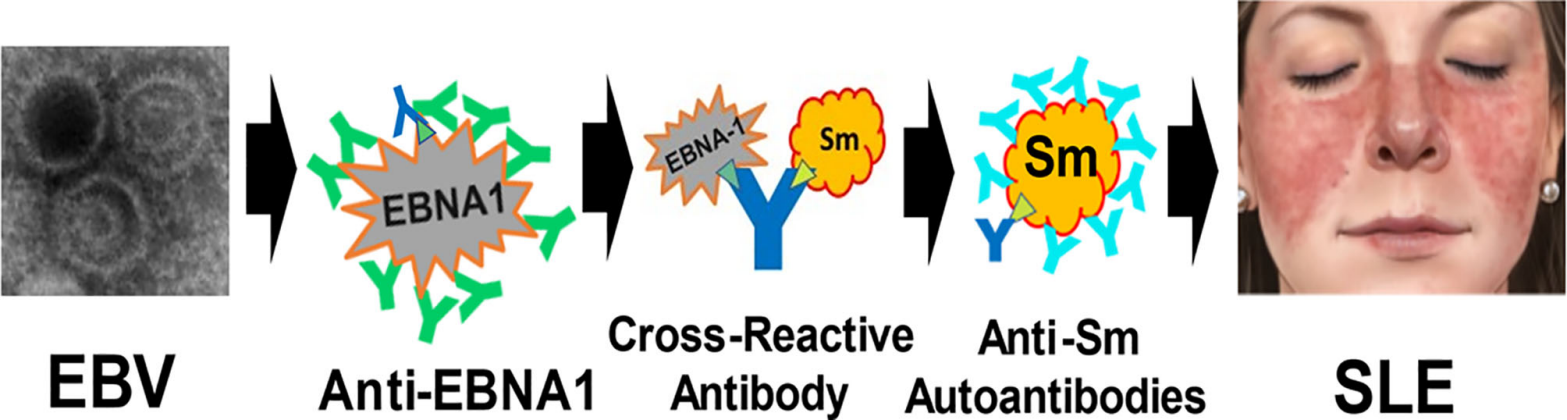
Model for the generation of systemic lupus erythematosus (SLE) by Epstein–Barr virus (EBV). After infection, EBV establishes a lifelong infection sustained by EBV in latency usually expressing at least Epstein–Barr nuclear antigen 1 (EBNA1). Anti-EBNA1 antibodies are produced in response to the EBV infection (green and dark blue **Y**). Eventually, one or more cross-reacting antibodies form (dark blue **Y)**, binding both EBNA1 and lupus autoantigens (e.g., Sm B/B’) at structures that are antigenically similar (green triangle). This is the molecular mimicry step with heteroimmune antibodies making the transition to autoimmunity. Then, B-cell epitope spreading leads to a mature complex autoantibody (lighter blue **Y**) response, inducing the inflammatory changes that culminate in the systemic disease manifestations of SLE. The specific role of EBV transcription cofactors [e.g., EBNA2, not shown (22)] and other contributing components in this model of SLE pathogenesis remains to be defined. (The EBV image, courtesy: National Institute of Allergy and Infectious Diseases. The SLE malar rash image, courtesy: Mayo Foundation, all rights reserved).

Herein, we confirm the hypothesis that the anti-EBNA1 heteroimmune response has the potential to be a critical component in the immunological sequence of events that culminate in SLE. This realization leads to a model of pathogenesis (**Figure 1**) in which EBV infection is followed by a polyclonal anti-EBNA1 heteroimmune response. As antibodies produced against EBNA1 increase in complexity through B-cell epitope spreading (25), some then participate in a cross-reaction against SLE autoantigens. Perhaps, considerations of stoichiometry, antigen concentration, and the peculiar properties of EBNA1 as an antigen are important at this point on the path toward SLE. EBNA1 is expressed in the latently EBV-infected B cells, which are rare, while the SLE autoantigens are ordinarily expressed ubiquitously. The CD8 T-cell anti-EBNA1 response is relatively inhibited, which would appear to help perpetuate the EBV infection. The molecular details of the mechanism leading to the cross-reactions between SLE autoantigens and EBNA1 are not known nor are the immunological rules that allow tolerance to be broken and autoantibodies to form in this context.

Our earlier work establishes the cross-reacting peptides for the autoantigens Sm and Ro as being the first structures of the autoantibody response (9, 12). Over time, B-cell epitope spreading then generates a mature autoimmune response, which in the case of SmB/B’ tends to recognize identical peptide structures in different SLE patients (8, 9) but for anti-60 kD Ro tends to be much more heterogeneous and to have a strong conformational contribution to antigenicity (29–31). For anti-Ro, autoantibody responses are often present many years (>5 years) before the onset of SLE symptoms, while anti-Sm tends to appear much closer to the onset of symptoms (32). After the generation of pathological autoantibodies in the proposed model, along with the other components of cellular and innate immunity supporting inflammatory pathology, the patient then becomes ill with the autoimmune disease SLE (**Figure 1**).

Ascertainment bias has an impact on the conclusions that can be drawn from these data. The entry criterion in Cui et al. (1) was whether the responsible physician ordered EBV serology. Hence, neither the SLE cases nor the controls can be considered authentic samples from the community. Nevertheless, distortions from the expected population norms are not apparent and, therefore, do not invalidate the results of the analysis. For example, EBV infection is known to be associated with SLE, which is also present in these data. Also, about 80% of 5–10-year-old Chinese children are EBV-infected (33, 34), which is close to results from the non-SLE controls in Cui et al. (1).

On the other hand, this study has the advantage of being based upon the choices of a real-world practitioner standard of care and not distorted by requiring SLE patients to satisfy academic criteria for study inclusion. These results are, nevertheless, consistent with the academic inquiries into SLE epidemiology, being robust for actual clinical practice in this regard and here *vice versa*.

Furthermore, none of the biases that originate from how the dataset was assembled is suspected to influence the central purpose of this study, to test the prediction that the proportion of SLE cases with anti-EBNA1 IgG would be higher than that found in controls matched for age, sex, and EBV infection status as measured by anti-VCA IgG antibodies. That we find a strong association supporting this prediction, even considering possible biases, suggests that the hypothesis that SLE autoimmunity arises from the anti-EBNA1 heteroimmune response is worth serious consideration and detailed experimental inquiry into plausible immune mechanisms.

Many studies have observed an association with anti-EBNA1 in SLE (1–7, 14, 23), but they have not, with one exception (14), attempted to determine any additional independent contribution anti-EBNA1 provides to SLE risk beyond EBV infection. In that small pediatric study composed of European-Americans and African-Americans, all the SLE cases were EBV-infected and produced anti-EBNA1, while almost a third of the EBV-infected controls did not have detectable levels of anti-EBNA1 (OR = 30.4, p < 0.001) (14). The combined results of the earlier study and the data presented herein show that the anti-EBNA1 heteroimmune response is a risk factor for SLE beyond EBV infection. On the one hand, anti-EBNA1 antibodies make an independent contribution to SLE risk, but on the other hand, they are a dependent risk factor in that only EBV-infected individuals generate anti-EBNA1 antibodies. Therefore, isolating anti-EBNA1 to assess it as a possible risk factor, separate from the association with EBV infection, is critical. We do this by considering an association with anti-EBNA1 in only those cases and controls who are EBV-infected. Our conclusion that anti-EBNA1 is an independent risk factor, only occurring in EBV-infected individuals and therefore also dependent upon EBV infection, bolsters the argument that EBV infection is etiologic in SLE.

Our results from both this and the previous study (14) also suggest that the presence of anti-EBNA1 may be a general finding in SLE with ~90% attributable fraction, therefore further supporting EBNA1 immunity being an important candidate for the origin of SLE autoimmunity. Collectively, these considerations provide the broad outlines of a model of SLE pathological generation, progressing from a normal immune response to a systemic life-threatening autoimmune disease (**Figure 1**).

There is another recent evidence potentially implicating EBV in generating SLE. The association of SLE genetic risk loci with the DNA-binding patterns of EBNA2 (35–37), a virus-encoded transcriptional cofactor required for B-cell transformation in the Latency III state of EBV expression and generating EBV-transformed B cells is consistent with EBNA2 being involved in the genomic regulation that alters the risk of developing SLE. This observation was originally established in Europeans and has since been independently confirmed twice in East Asians (35–37), providing consistent findings across the major human ancestries. This association, by itself however, does not require a pathogenic role for EBNA2. There are other suggestive results increasing the circumstantial evidence that EBV infection contributes to the propensity for autoimmunity, especially with the latency expression programs of EBV infection. For example, the CD40-imitating capacity of LMP1 (latent membrane protein 1), an EBV gene product expressed in latency, appears to lower the threshold for humoral autoimmunity (38). However, when these observations are combined with the immunochemistry and epidemiological observations concerning autoantibody origin, the association of SLE with EBV infection, and the added risk from anti-EBNA1, the accumulated EBV-related evidence begins to nominate possible molecular components of mechanism and to provide a plausible outline of a mechanistic scenario for EBV action starting with EBV infection and progressing to the clinical manifestations of SLE (**Figure 1**).

The fraction of SLE attributable to EBV in this sample, using the matching strategy to remove the effects of sex and age, is complete at 100% and is consistent with a previous similarly high estimate (2). Together, they suggest that EBV infection contributes to SLE in the great majority of SLE patients, while major single gene causes of SLE such as C1q or TREX1 deficiencies are uncommon. While useful for understanding the mechanism to establish that EBV is the likely source for the pathophysiology in initiating the process culminating in SLE, EBV infection, by itself, does not much distinguish risk for SLE from the vast majority of the population that are also EBV-infected (>90% worldwide) but not afflicted with SLE. Consequently, other factors must contribute. Certainly, the collective vagaries of the mature host immune response are strong candidates for SLE disease risk. Our analyses combined with other contributing results would suggest that a molecular mimicry mechanism making the transition from heteroimmunity to autoimmunity contributes to the pathophysiology of SLE (**Figure 1**).

That SLE autoimmunity emerges from the anti-EBNA1 heteroimmune response came from an effort to work backward temporally. We began with the complex antigenic epitopes of the mature pathogenic SLE autoantibody responses, then reached back in time to find the simple, single, earliest antigenic autoimmune epitope. The goal was to discover the first initiating autoantibody that identifies the first autoimmune structure in SLE (9, 12). For both the Sm B/B’ and Ro SLE autoantigens, the earliest autoantibodies cross-reacted with EBNA1, leading to the hypothesis that anti-EBNA1 humoral immunity is the source of pathogenic SLE autoimmunity; hence, the prediction we test herein. Other investigators have developed data consistent with anti-EBNA1 being the origin of the anti-Sm D and anti-C1q autoimmune responses (10, 11, 14). If the hypothesis is indeed true that SLE autoimmunity has a strong tendency to originate from the anti-EBNA1 response, then one might suspect that other SLE autoantigens will eventually join these four.

A strong candidate SLE autoantibody that begs testing is anti-double-stranded (ds) DNA. Linda Spatz has shown that immunizing animals with EBNA1 generates anti-dsDNA antibodies (39–41), but to our knowledge, no one has evaluated whether the anti-dsDNA autoantibodies, which are almost unique to SLE and powerfully support an SLE diagnosis when present, also cross-react with EBNA1. The fact that EBNA1 is a DNA-binding protein raises interesting idiotype and anti-idiotype issues that await exploration.

Our results clearly show that anti-EBNA1 IgG is present in virtually all (>99%) SLE patients, providing an origin for SLE in as many as 90% of SLE cases (**Table 1**) and supporting the contention that EBV is the ordinary causal factor in the great majority of SLE cases. These results confirm the importance of EBV infection in SLE and provide a starting point for explaining the mysterious mechanistic steps that lead previously normal individuals to develop pathogenic SLE autoantibodies and self-destructive clinical manifestations.

Our model of pathogenesis presents a certain sequence of events in the following order: EBV infection, anti-EBNA1 antibodies, SLE autoantibodies, and pathogenic expression of disease. In the military serum collection (32), there are four SLE patients whose multiple serum samples show exactly this sequence of events. There are 17 SLE patients in this collection from whom there were sera available that discriminated the onset of anti-EBNA1 from SLE autoantibodies. In all 17 SLE cases, the anti-EBNA1 antibodies were detected in an earlier serum sample than was any SLE autoantibody. In no instance did autoantibodies precede anti-EBNA1 antibodies (p < 0.00002, binomial test). These data also support the model being proposed (**Figure 1**). While available data support the outlines of a proposed mechanism (**Figure 1**), we are missing the detailed cellular and molecular mechanism for how these steps lead to SLE. While the unusual T-cell antigenicity of EBNA1 has been subject of detailed inquiry (16–22), the humoral B-cell side of EBNA1 antigenicity has not.

The finding that EBNA2, not EBNA1, is associated with the risk loci of SLE (35–37) provides an important potential candidate for mechanistic involvement that increases the risk of developing SLE in genetically predisposed individuals and suggests that the cell types that harbor the EBV [infected B cells, T cells, natural killer (NK) cells, epithelial cells, and macrophages] would be the most attractive candidates for the cell types in which these mechanisms operate to alter the SLE risk. Such factors provide access to gene and environment interactive mechanisms that, once understood, may prove to place EBV infection and anti-EBNA1 antibodies in mechanistic context.

The increased anti-VCA IgA response is consistent with the mucosal interaction with EBV being more important in SLE patients than controls. As a result of its prevalence, anti-EBNA1 IgG would appear to be a better candidate for initiation of SLE autoimmunity than is anti-VCA IgA. Moreover, the increase in anti-VCA IgA is a relative result, and the fraction attributable to this immune response at 74.4% is much lower than it is with the anti-EBNA1 IgG humoral immune response at 89.7%.

In summary, the Cui et al. (1) data further confirm the association of SLE with EBV and extend these findings to East Asians. Matched analysis confirms the importance of anti-EBNA1 responses in SLE, consistent with this heteroimmune response being important in the origin of autoimmunity in SLE. Other data assembled show high EBV viral loads, increased EBV mRNA expression, elevated humoral responses against EBV in SLE (3–5), and the concentration of EBNA2 at SLE risk loci (35–37), all supporting a model of SLE pathogenesis involving EBV with SLE patients having an altered infection pattern with poor control of the latent EBV infection and incomplete EBV lytic reactivation and providing a synergistic environmental interaction with genetic risk loci through EBNA2. While there is much to learn concerning the mechanisms that generate the systemic autoimmunity in SLE, we are left with the conclusion that EBV, probably through EBNA1, is a strong candidate to be the initiating source for the autoimmune processes that culminate in SLE.

## Data Availability Statement

The original contributions presented in the study are included in the article/**Supplementary Material**. Further inquiries can be directed to the corresponding author.

## Author Contributions

JH conceived the study. VL, LD, and JH designed the study with input from KK and JJ. VL organized the database and performed the case–control matching. LD and VL led the statistical analysis aided by JH. JH, VL, and LD drafted the article with revisions and conceptual insights from KK and JJ. All authors contributed to the article and approved the submitted version.

## Funding

Generous financial and institutional support in our quest to explain SLE pathogenesis is appreciated including NIH grants R01 AI024717, R01 AI148276, U01 AI130830, UM1 AI144292, U54 GM104938, and P30 AR073750 and a USDVA Merit Award I01 BX001834.

## Conflict of Interest

The authors declare that the research was conducted in the absence of any commercial or financial relationships that could be construed as a potential conflict of interest.

## Publisher’s Note

All claims expressed in this article are solely those of the authors and do not necessarily represent those of their affiliated organizations, or those of the publisher, the editors and the reviewers. Any product that may be evaluated in this article, or claim that may be made by its manufacturer, is not guaranteed or endorsed by the publisher.
