# Supplementary material for: A High Prevalence of Anti-EBNA1 Heteroantibodies in Systemic Lupus Erythematosus (SLE) Supports Anti-EBNA1 as an Origin for SLE Autoantibodies
Source: Front Immunol. 2022 Feb 17;13:830993. doi: 10.3389/fimmu.2022.830993 (PMC8892314; doi:10.3389/fimmu.2022.830993)
Supplement: Supplementary file 2 [file Table_2.docx]

Supplementary Material

Table S2: Association between systemic lupus erythematosus (SLE) status and anti-EBV antibodies in age and sex matched cases and controls.

|  |  |  |  | Conditional Logistic Regression^a^ | | | Logistic Regression^e^ | | | |
| --- | --- | --- | --- | --- | --- | --- | --- | --- | --- | --- |
| Anti-EBV Antibodies |  | SLE | Controls | OR^b^  (95%CI) | p | Attributable  Fraction –  OR (%)^d^ | OR  (95%CI) | p | Attributable  Fraction – OR (%)^f^ | Attributable  Fraction –  RR (%)^g^ |
| Anti-VCA IgG | POS | 232 | 642 | 28.59  (6.42, ∞) | 8.8×10^-8 c^ | 100 | 39.45  (2.37, 657.99) | 0.0105 | 97.5  (90.3, 100) | 96.6  (87.0, 100) |
|  | NEG | 0 | 54 |  |  |  |  |  |  |  |
|  | | | | | | | | | | |
| Anti-EBNA1 IgG | POS | 124 | 217 | 19.07  (3.09, 789.48) | 1.8×10^-5 c^ | 94.8  (84.2, 100) | 12.79  (2.40, 68.25) | 0.0029 | 92.2  (79.1, 100) | 88.2  (69.5, 100) |
|  | NEG | 1 | 33 |  |  |  |  |  |  |  |
|  | | | | | | | | | | |
| Anti-EBNA1 IgG in  Anti-VCA IgG POS | POS | 124 | 232 | 9.74  (1.49, 414.34) | 0.0078^c^ | 89.7  (68.9, 100) | 6.61  (1.18, 36.99) | 0.0315 | 84.9  (58.8, 100) | 78.5  (44.4, 100) |
|  | NEG | 1 | 18 |  |  |  |  |  |  |  |
|  | | | | | | | | | | |
| Anti-VCA IgA in  Anti-VCA IgG POS | POS | 72 | 56 | 3.47  (2.27, 5.29) | 5.7×10^-8^ | 71.2  (59.0, 83.3) | 3.90  (2.55, 5.98) | 4.0×10^-10^ | 74.4  (63.4, 85.3) | 55.9  (45.4, 66.5) |
|  | NEG | 85 | 258 |  |  |  |  |  |  |  |

^a^ Exact conditional logistic regression was used when some of the cells formed by the presence of anti-EBV antibodies and SLE status had no observations or were too small for a reliable conditional logistic regression analysis.

^b^ Odds ratio (OR) of SLE comparing positive vs. negative anti-EBV antibodies.

^c^ Indicates a median unbiased estimate and a one-sided p-value from exact conditional logistic regression models.

^d^ Attributable Fraction of exposure of anti-EBV antibodies estimated based on odds ratio (OR) estimated from exact conditional logistic regression: i.e., Attributable Fraction =(OR-1)/OR.

^e^ Firth logistic regression was used when some of the cells formed by the presence of anti-EBV antibodies and SLE status had no observations or were too small.

^f^ Attributable Fraction of exposure of anti-EBV antibodies estimated based on OR estimated from logistic regression: i.e., Attributable Fraction=(OR-1)/OR.

^g^ Attributable Fraction of exposure of anti-EBV antibodies estimated based on risk ratio (RR) estimated from logistic regression: i.e., Attributable Fraction=(RR-1)/RR.

Table S3: Association between systemic lupus erythematosus (SLE) status and anti-EBV antibodies in age and sex matched cases and controls (6-10 years old).

|  |  |  |  | Conditional Logistic Regression^a^ | | | Logistic Regression^e^ | | | |
| --- | --- | --- | --- | --- | --- | --- | --- | --- | --- | --- |
| Anti-EBV Antibodies |  | SLE | Controls | OR^b^  (95%CI) | p | Attributable  Fraction –  OR (%)^d^ | OR  (95%CI) | p | Attributable  Fraction – OR (%)^f^ | Attributable  Fraction –  RR (%)^g^ |
| Anti-VCA IgG | POS | 52 | 133 | 12.68  (2.71, ∞) | 0.0008^c^ | 100 | 18.49  (1.04, 329.10) | 0.0471 | 94.6  (79.0, 100) | 92.6  (71.9, 100) |
|  | NEG | 0 | 23 |  |  |  |  |  |  |  |
|  | | | | | | | | | | |
| Anti-EBNA1 IgG | POS | 33 | 75 | 14.07  (2.99, ∞) | 0.0005^c^ | 100 | 21.75  (1.21, 390.09) | 0.0366 | 95.4  (82.1, 100) | 93.5  (75.2, 100) |
|  | NEG | 0 | 24 |  |  |  |  |  |  |  |
|  | | | | | | | | | | |
| Anti-EBNA1 IgG in  Anti-VCA IgG POS | POS | 33 | 61 | 3.36  (0.61, ∞) | 0.1317^c^ | 100 | 6.00  (0.24, 147.14) | 0.2727 | 83.3  (30.0, 100) | 76.4  (74.0, 100) |
|  | NEG | 0 | 5 |  |  |  |  |  |  |  |
|  | | | | | | | | | | |
| Anti-VCA IgA in  Anti-VCA IgG POS | POS | 65 | 17 | 6.76  (2.81, 16.24) | 2.0x10^-5^ | 85.2  (72.2, 98.2) | 5.97  (2.62, 13.62) | 2.1x10^-5^ | 83.3  (69.5, 97.1) | 66.8  (50.1, 83.6) |
|  | NEG | 16 | 65 |  |  |  |  |  |  |  |

^a^ Exact conditional logistic regression was used when some of the cells formed by the presence of anti-EBV antibodies and SLE status had no observations or were too small.

^b^ Odds ratio (OR) of SLE comparing positive vs. negative anti-EBV antibodies.

^c^ Indicates a median unbiased estimate and a one-sided p-value from exact conditional logistic regression models.

^d^ Attributable Fraction of exposure of anti-EBV antibodies estimated based on odds ratio (OR) estimated from exact conditional logistic regression: i.e., Attributable Fraction=(OR-1)/OR.

^e^ Firth logistic regression was used when some of the cells formed by the presence of anti-EBV antibodies and SLE status had no observations or were too small.

^f^ Attributable Fraction of exposure of anti-EBV antibodies estimated based on OR estimated from logistic regression: i.e., Attributable Fraction=(OR-1)/OR.

^g^ Attributable Fraction of exposure of anti-EBV antibodies estimated based on risk ratio (RR) estimated from logistic regression: i.e., Attributable Fraction=(RR-1)/RR.

Table S4: Association between systemic lupus erythematosus (SLE) status and anti-EBV antibodies in age and sex matched cases and controls (11 years and older).

|  |  |  |  | Conditional Logistic Regression^a^ | | | Logistic Regression^e^ | | | |
| --- | --- | --- | --- | --- | --- | --- | --- | --- | --- | --- |
| Anti-EBV Antibodies |  | SLE | Controls | OR^b^  (95%CI) | p | Attributable  Fraction –  OR (%)^d^ | OR  (95%CI) | p | Attributable  Fraction – OR (%)^f^ | Attributable  Fraction –  RR (%)^g^ |
| Anti-VCA IgG | POS | 180 | 509 | 15.64  (3.45, ∞) | 0.0001^c^ | 100 | 22.33  (1.30, 383.74) | 0.0323 | 95.5  (82.8, 100) | 94.0  (77.3, 100) |
|  | NEG | 0 | 31 |  |  |  |  |  |  |  |
|  | | | | | | | | | | |
| Anti-EBNA1 IgG | POS | 91 | 166 | 10.52  (1.56, 453.72) | 0.0063^c^ | 90.5  (71.0, 100) | 6.78  (1.21, 38.08) | 0.0298 | 85.3  (59.8, 100) | 78.9  (45.3, 100) |
|  | NEG | 1 | 18 |  |  |  |  |  |  |  |
|  | | | | | | | | | | |
| Anti-EBNA1 IgG in  Anti-VCA IgG POS | POS | 91 | 171 | 7.17  (0.91, 56.52) | 0.0462^c^ | 86.1  (57.3, 100) | 4.80  (0.82, 28.03) | 0.0814 | 79.2  (42.4, 100) | 71.3  (25.8, 100) |
|  | NEG | 1 | 13 |  |  |  |  |  |  |  |
|  | | | | | | | | | | |
| Anti-VCA IgA in  Anti-VCA IgG POS | POS | 47 | 39 | 3.04  (1.85, 4.99) | 1.1x10^-5 c^ | 67.1  (50.8, 83.4) | 3.37  (2.03, 5.59) | 2.5x10^-6^ | 70.3  (55.3, 85.3) | 51.8  (38.4, 65.3) |
|  | NEG | 69 | 193 |  |  |  |  |  |  |  |

^a^ Exact conditional logistic regression was used when some of the cells formed by the presence of anti-EBV antibodies and SLE status had no observations or were too small.

^b^ Odds ratio (OR) of SLE comparing positive vs. negative anti-EBV antibodies.

^c^ Indicates a median unbiased estimate and a one-sided p-value from exact conditional logistic regression models.

^d^ Attributable Fraction of exposure of anti-EBV antibodies estimated based on odds ratio (OR) estimated from exact conditional logistic regression: i.e., Attributable Fraction=(OR-1)/OR.

^e^ Firth logistic regression was used when some of the cells formed by the presence of anti-EBV antibodies and SLE status had no observations or were too small.

^f^ Attributable Fraction of exposure of anti-EBV antibodies estimated based on OR estimated from logistic regression: i.e., Attributable Fraction=(OR-1)/OR.

^g^ Attributable Fraction of exposure of anti-EBV antibodies estimated based on risk ratio (RR) estimated from logistic regression: i.e., Attributable Fraction=(RR-1)/RR.

Table S5: Association between systemic lupus erythematosus (SLE) status and anti-EBV antibodies – unmatched data.

|  |  |  |  | Logistic Regression^b^ | | | |
| --- | --- | --- | --- | --- | --- | --- | --- |
|  |  | SLE | Controls | OR^a^  (95%CI) | p | Attributable  Fraction – OR (%)^c^ | Attributable  Fraction – RR (%)^d^ |
|  |  |  |  |  |  |  |  |
| Anti-VCA IgG (n) | POS | 232 | 5396 | 45.46 (2.78, 744.48) | 0.0075 | 97.8 (91.7, 100) |  |
|  | NEG | 0 | 175 |  |  |  |  |
| Sex (n) | Female | 199 | 2794 | 6.02 (4.13, 8.75) | 6.5x10^-21^ |  | 97.7 (91.4, 100) |
|  | Male | 33 | 2777 |  |  |  | 97.8 (91.6, 100) |
| Age, mean (sd) |  | 20.0 (14.0) | 39.5 (19.9) | 0.93 (0.92, 0.94) | <1.0x10^-24^ |  |  |
|  | | | | | | | |
| Anti-EBNA1 IgG | POS | 124 | 1403 | 17.58 (3.42, 90.38) | 0.0006 | 94.3 (85.0, 100) |  |
|  | NEG | 1 | 152 |  |  |  |  |
| Sex (n) | Female | 110 | 796 | 6.87 (3.96, 11.90) | 6.5x10^-12^ |  | 93.8 (83.8, 100) |
|  | Male | 15 | 759 |  |  |  | 94.2 (84.8, 100) |
| Age, mean (sd) |  | 16.6 (11.9) | 34.4 (22.0) | 0.94 (0.93, 0.95) | 1.3x10^-15^ |  |  |
|  | | | | | | | |
| Anti-EBNA1 IgG in  Anti-VCA IgG POS | POS | 124 | 1399 | 8.62 (1.64, 45.36) | 0.0110 | 88.4 (69.2, 100) |  |
|  | NEG | 1 | 89 |  |  |  |  |
| Sex (n) | Female | 110 | 763 | 6.79 (3.92, 11.77) | 8.6x10^-12^ |  | 87.6 (67.1, 100) |
|  | Male | 15 | 725 |  |  |  | 88.3 (68.8, 100) |
| Age, mean (sd) |  | 16.6 (11.9) | 35.3 (21.9) | 0.94 (0.93, 0.95) | 1.1x10^-15^ |  |  |
|  | | | | | | | |
| Anti-VCA IgA in  Anti-VCA IgG POS | POS | 72 | 1108 | 2.54 (1.80, 3.60) | 1.4x10^-7^ | 60.7 (47.0, 74.3) |  |
|  | NEG | 85 | 2592 |  |  |  |  |
| Sex (n) | Female | 137 | 1846 | 6.59 (4.07, 10.67) | 1.9x10^-14^ |  | 59.5 (45.9, 73.0) |
|  | Male | 20 | 1854 |  |  |  | 60.5 (46.8, 74.1) |
| Age, mean (sd) |  | 17.3 (12.2) | 39.0 (19.4) | 0.92 (0.91, 0.94) | <1.0x10^-24^ |  |  |

^a^ Odds ratio (OR) of SLE comparing positive vs. negative anti-EBV antibodies; female vs. male; and for every 1-year increase in age.

^b^ Logistic regression with adjustment of age and sex. Firth logistic regression was used when some of the cells formed by the presence of anti-EBV antibodies and SLE status had no observations or were too small.

^c^ Attributable Fraction of exposure of anti-EBV antibodies estimated based on OR estimated from logistic regression: i.e., Attributable Fraction=(OR-1)/OR.

^d^ Attributable Fraction of exposure of anti-EBV antibodies estimated based on risk ratio (RR) estimated from logistic regression: i.e., Attributable Fraction=(RR-1)/RR. Since female and male had different RR, Attributable Fraction was estimated by sex.

Table S6: Association between systemic lupus erythematosus (SLE) status and anti-EBV antibodies (6-10 years old) – unmatched data.

|  |  |  |  | Logistic Regression^b^ | | | |
| --- | --- | --- | --- | --- | --- | --- | --- |
|  |  | SLE | Controls | OR^a^  (95%CI) | p | Attributable  Fraction – OR (%)^c^ | Attributable  Fraction – RR (%)^d^ |
|  |  |  |  |  |  |  |  |
| Anti-VCA IgG (n) | POS | 52 | 379 | 22.75 (1.38, 373.95) | 0.0287 | 95.6 (83.3, 100) |  |
|  | NEG | 0 | 80 |  |  |  |  |
| Sex (n) | Female | 45 | 229 | 6.22 (2.81, 13.76) | 6.5x10^-6^ |  | 94.6 (79.7, 100) |
|  | Male | 7 | 230 |  |  |  | 95.4 (82.7, 100) |
| Age, mean (sd) |  | 8.5 (1.3) | 8.1 (1.5) | 1.17 (0.95, 1.45) | 0.1356 |  |  |
|  | | | | | | | |
| Anti-EBNA1 IgG | POS | 33 | 184 | 23.06 (1.38, 384.78) | 0.0289 | 95.7 (83.5, 100) |  |
|  | NEG | 0 | 57 |  |  |  |  |
| Sex (n) | Female | 29 | 125 | 7.09 (2.52, 19.91) | 0.0002 |  | 94.4 (78.9, 100) |
|  | Male | 4 | 116 |  |  |  | 95.5 (82.8, 100) |
| Age, mean (sd) |  | 8.6 (1.1) | 8.0 (1.5) | 1.33 (0.995, 1.78) | 0.0538 |  |  |
|  | | | | | | | |
| Anti-EBNA1 IgG in  Anti-VCA IgG POS | POS | 33 | 182 | 8.05 (0.43, 147.93) | 0.1604 | 87.6 (51.4, 100) |  |
|  | NEG | 0 | 21 |  |  |  |  |
| Sex (n) | Female | 29 | 104 | 6.86 (2.44, 19.28) | 0.0003 |  | 84.3 (40.6, 100) |
|  | Male | 4 | 99 |  |  |  | 87.1 (49.7, 100) |
| Age, mean (sd) |  | 8.6 (1.1) | 8.0 (1.5) | 1.34 (0.999, 1.79) | 0.0511 |  |  |
|  | | | | | | | |
| Anti-VCA IgA in  Anti-VCA IgG POS | POS | 25 | 59 | 4.86 (2.38, 9.90) | 1.4x10^-5^ | 79.4 (64.7, 94.1) |  |
|  | NEG | 16 | 228 |  |  |  |  |
| Sex (n) | Female | 36 | 147 | 5.26 (1.96, 14.08) | 0.0010 |  | 70.8 (54.5, 87.2) |
|  | Male | 5 | 140 |  |  |  | 77.6 (62.7, 92.6) |
| Age, mean (sd) |  | 8.4 (1.3) | 8.2 (1.5) | 1.12 (0.87, 1.44) | 0.3855 |  |  |

^a^ Odds ratio (OR) of SLE comparing positive vs. negative anti-EBV antibodies; female vs. male; and for every 1-year increase in age.

^b^ Logistic regression with adjustment of age and sex. Firth logistic regression was used when some of the cells formed by the presence of anti-EBV antibodies and SLE status had no observations or too small.

^c^ Attributable Fraction of exposure of anti-EBV antibodies estimated based on OR estimated from logistic regression: i.e., Attributable Fraction =(OR-1)/OR.

^d^ Attributable Fraction of exposure of anti-EBV antibodies estimated based on risk ratio (RR) estimated from logistic regression: i.e., Attributable Fraction=(RR-1)/RR. Since female and male had different RR, Attributable Fraction was estimated by sex.

Table S7: Association between systemic lupus erythematosus (SLE) status and anti-EBV antibodies (11 years and older) – unmatched data.

|  |  |  |  | Logistic Regression^b^ | | | |
| --- | --- | --- | --- | --- | --- | --- | --- |
|  |  | SLE | Controls | OR^a^  (95%CI) | p | Attributable  Fraction – OR (%)^c^ | Attributable  Fraction – RR (%)^d^ |
|  |  |  |  |  |  |  |  |
| Anti-VCA IgG (n) | POS | 180 | 5017 | 17.86 (1.07, 299.20) | 0.0451 | 94.4 (78.6, 100) |  |
|  | NEG | 0 | 95 |  |  |  |  |
| Sex (n) | Female | 154 | 2565 | 5.85 (3.84, 8.92) | 1.9x10^-16^ |  | 94.3 (78.1, 100) |
|  | Male | 26 | 2547 |  |  |  | 94.4 (78.5, 100) |
| Age, mean (sd) |  | 23.3 (14.3) | 42.3 (18.3) | 0.93 (0.91, 0.94) | <1.0x10^-24^ |  |  |
|  | | | | | | | |
| Anti-EBNA1 IgG | POS | 91 | 1219 | 7.30 (1.39, 38.30) | 0.0188 | 86.3 (63.6, 100) |  |
|  | NEG | 1 | 95 |  |  |  |  |
| Sex (n) | Female | 81 | 671 | 6.44 (3.41, 12.17) | 9.7x10^-9^ |  | 85.6 (61.9, 100) |
|  | Male | 11 | 643 |  |  |  | 86.2 (63.3, 100) |
| Age, mean (sd) |  | 19.5 (12.7) | 39.2 (20.6) | 0.93 (0.91, 0.95) | 2.8x10^-14^ |  |  |
|  | | | | | | | |
| Anti-EBNA1 IgG in  Anti-VCA IgG POS | POS | 91 | 1217 | 4.44 (0.83, 23.88) | 0.0824 | 77.5 (39.6, 100) |  |
|  | NEG | 1 | 68 |  |  |  |  |
| Sex (n) | Female | 81 | 659 | 6.40 (3.39, 12.11) | 1.1x10^-8^ |  | 76.5 (37.4, 100) |
|  | Male | 11 | 626 |  |  |  | 77.3 (39.3, 100) |
| Age, mean (sd) |  | 19.5 (12.7) | 39.6 (20.5) | 0.93 (0.91, 0.95) | 2.5x10^-14^ |  |  |
|  | | | | | | | |
| Anti-VCA IgA in  Anti-VCA IgG POS | POS | 47 | 1049 | 2.09 (1.39, 3.14) | 0.0004 | 52.2 (32.8, 71.6) |  |
|  | NEG | 69 | 2364 |  |  |  |  |
| Sex (n) | Female | 101 | 1699 | 6.79 (3.89, 11.83) | 1.5x10^-11^ |  | 51.4 (32.2, 70.6) |
|  | Male | 15 | 1714 |  |  |  | 52.1 (32.7, 71.4) |
| Age, mean (sd) |  | 20.4 (12.7) | 41.6 (18.0) | 0.91 (0.90, 0.93) | <1.0x10^-24^ |  |  |

^a^ Odds ratio (OR) of SLE comparing positive vs. negative anti-EBV antibodies; female vs. male; and for every 1-year increase in age.

^b^ Logistic regression with adjustment of age and sex. Firth logistic regression was used when some of the cells formed by the presence of anti-EBV antibodies and SLE status had no observations or too small.

^c^ Attributable Fraction of exposure of anti-EBV antibodies estimated based on OR estimated from logistic regression: i.e., Attributable Fraction=(OR-1)/OR.

^d^ Attributable Fraction of exposure of anti-EBV antibodies estimated based on risk ratio (RR) estimated from logistic regression: i.e., Attributable Fraction=(RR-1)/RR. Since female and male had different RR, Attributable Fraction was estimated by sex.
